# Cooling Ability/Capacity and Exergy Penalty Analysis of Each Heat Sink of Modern Supersonic Aircraft

**DOI:** 10.3390/e21030223

**Published:** 2019-02-26

**Authors:** Yu-Feng Mao, Yun-Ze Li, Ji-Xiang Wang, Kai Xiong, Jia-Xin Li

**Affiliations:** 1 School of Aeronautic Science and Engineering, Beihang University, Beijing 100191, China; 2 Advanced Research Center of Thermal and New Energy Technologies, Xingtai Polytechnic College, Xingtai 054035, China; 3 Institute of Engineering Thermophysics, North China University of Water Conservancy and Electric Power, Zhengzhou 450045, China; 4 School of Automation Science and Electrical Engineering, Beihang University, Beijing 100191, China

**Keywords:** aircraft, thermal management system, heat sink, cooling ability, cooling capacity, exergy

## Abstract

The aerospace-based heat sink is defined as a substance used for dissipating heat generated by onboard heat loads. They are becoming increasingly scarce in the thermal management system (TMS) of advanced aircraft, especially for supersonic aircraft. In the modern aircraft there are many types of heat sinks whose cooling abilities and performance penalties are usually obviously different from each other. Besides, the cooling ability and performance penalty of a single heat sink is even different under different flight conditions—flight altitude, Mach number, etc. In this study, the typical heat sinks which are the fuel mass, ram air, engine fan air, skin heat exchanger, and expendable heat sink will be studied. Their cooling abilities/capacities, and exergy penalties under different flight conditions have been systematically estimated and compared with each other. The exergy penalty presented in this paper refers to the exergy loss of aircraft caused by the extra weight, drag and energy extraction of various heat sinks. The estimation models, as well as the results and discussion have been elaborated in this paper, which can be can be used to further optimize the TMS of modern advanced aircraft, for example, the layout design of various heat sinks and the improvement the control algorithm.

## 1. Introduction

Nowadays, modern advanced aircraft suffer from severe thermal management issues caused by the reduction in the cooling ability of traditional heat sinks and the increase in the head load of the aircraft itself [1,2]. Due to the increasing flight altitude and Mach number, the cooling ability of traditional heat sinks is reduced significantly [3]. The increasing Mach number will lead to a rapid rise in the total/stagnation temperature of ambient heat sinks such as ram air (RA) and fuselage skin, and the high altitude will result in rarefied atmosphere which could make the source of the heat sink less available. Besides, the aerodynamic shape and high stealth of the modern advantage aircraft also make it even more difficult to obtain ambient heat sinks, resulting in a serious decrease in their cooling ability [4]. The decrease in ambient heat sinks’ cooling ability will cause an increasing dependence on the fuel heat sink [5]. However, the cooling capability of fuel is restrictively limited by its residual mass in tank, which will become very scarce in the later stages of the flight period because of the constant consumption of fuel. The fuel’s increasing temperature affects this too. What’s worse, the heat load level of advanced aircrafts has been enhanced rapidly [6]. Three primary reasons might be responsible for such vigorous enhancement. Firstly, it is reported that the overall avionics heat load of the fourth generation aircraft such as the Lockheed Martin F22 reaches over 100 kW [7,8]. Secondly, the aerodynamic heating of a hypersonic vehicle, as mentioned above, will heat the fuselage structure to an extreme temperature, for example, it can exceed 2200 °C on the surface at 5 Ma [9,10], further intensifying the overall heat load. Thirdly, advanced on-board electronic and electrical devices such as the large phased array radar [11] and laser weapons [12,13] are highly integrated and micro-miniaturized [14], where the instantaneous heat load can be up to MW level [15]. Therefore, the gap between the cooling capacity of the heat sink and the on-board heat load must be narrowed for the reliable and efficient operation of the aircraft.

The thermal management system (TMS) is one of the subsystems of aircraft, which utilizes various heat sinks to cool the thermal loads, maintaining a safe and efficient working environment for airborne equipment. The main part of the TMS of modern advanced aircraft is the fuel cooling circuit that is known as the fuel TMS [16,17,18]. The fuel collects heat from various thermal loads while flowing through the heat exchangers (HX), then it is burnt in the engine or goes back to the tank. Other heat sinks such as RA, engine fan air (EFA), skin heat exchanger (SHX), can be integrated into the TMS by various layout methods to cool the thermal loads directly or indirectly [19,20]. To narrow the gap presented in the first paragraph on the basis of typical TMS in modern advantaged aircraft, two possible methods have been mainly presented by researchers: increasing the cooling ability of the heat sinks and optimizing the TMS for more efficient usage of each available heat sink. These two methods will be described in detail in the following two paragraphs.

First, many efforts have been devoted to increasing the cooling ability of heat sinks. The endothermic process of thermal cracking is an effective way to improve the heat sink capacity of fuel [21,22]. Hou et al. [23] investigated the heat transfer and thermal cracking behavior both experimentally and numerically. Wickham et al [24] increased the rate of thermal cracking reactions by using a fuel additive which allows a similar chemical endothermic process to be obtained at lower fuel temperatures. Besides, the third bypass stream presented in the adaptive cycle engine for the next generation fighter, is another potential method to solve the problem of a lack of heat sinks [25,26], which can provide ample bypass air as heat sink for the TMS [27]. In recent years, expendable heat sinks (EHSs) such as endothermic chemical reactions and liquid gases which can absorb heat at normal/low temperature (below the water freezing point) have attracted more and more attention. Johnson et al. [28,29] developed a controllable expendable TMS, which uses the endothermic chemical reaction of ammonium carbamate and propylene glycol as heat sink. Nuzum et al. [30,31] presented a cryogenic-based TMS in which the liquefied natural gas (LNG) was used to thermally manage the high energy pulsed systems under low temperature, and the LNG can also be burned in a power plant to generate power for the aircraft energy system. 

The second way to optimize the TMS is to improve the thermal management efficiency through more rational structure, more efficient heat transfer method or advanced control strategies/ algorithms. The improvement of HX is one of the effective methods, which cannot only improve the thermal management ability but also reduce the weight of a TMS [32,33]. Similarly, the improvement of fuel pumps is another effective method: a “variable speed pump system” has been proposed by Daisuke and Yukinori [34] which can reduce the energy consumption of the fuel recirculation system. Besides, several advanced heat transfer methods of TMS have also been proposed. Wang et al. [35,36,37] investigated the flash boiling spray cooling under the high altitude condition (low ambient pressure), which shows excellent heat dissipation ability for extreme heat flux devices. Deng [38] and Ma [39] have investigated the mini-channel heat sink numerically and experimentally, showing good performance for the application in the cooling of high heat flux microelectronic devices. Phase change materials for the thermal protection of onboard electrical motors have also been investigated numerically and experimentally [40,41], indicating that it is a good thermal control method for drastically changing heat loads. In addition, advanced thermal managing strategies and control algorithm are effective research fields. Jain et al. [42] presented an exergy-based objective function to solve the multi-objective optimal control problems for TMS. Bodie et al. [43] developed a robust optimization for reducing the effect of uncertainty on TMS, in which three factors are comprehensively considered: EFA temperature, avionics thermal load, and engine thrust. Deppen et al. [44] proposed a predictive control strategy which utilizes preview of upcoming loads and disturbances to prevent violation of temperature constraints. 

Studies presented in the two above paragraphs suggest two ways to improve the TMS: obtaining more available sources of the heat sink and making the TMS more efficient. No matter which way is considered, the heat sink is the key. Typical heat sinks for airborne vehicles can be divided into two main types [19]: fuel and ambient heat sinks. The latter ones can be divided into RA, EFA, and SHX. In addition, the EHS has attracted more and more attention these years [45].

There are big differences between various heat sinks in terms of cooling ability/capacity and the performance penalty, both of which are important for optimizing the design of each heat sink in the TMS. Firstly, the cooling ability of each heat sink is different, and different heat sinks have different operating characteristics. For example, the fuel can provide sufficient cooling capacity but it is limited by the upper temperature limit and the EHS can provide low-temperature cooling [46,47]. In addition, the cooling ability of one heat sink under different flight conditions will be obviously different. For example, the temperature of the ambient heat sinks generally increases with the increase of Mach number, leading to a decrease in its cooling ability [48,49]. Secondly, the performance penalty of each heat sink is also different with each other. Generally, the performance penalty of the ancillary equipment in the TMS should be defined as the fuel mass penalty which is benefit to the early design and optimization of the aircraft. About the determination of fuel mass penalty, the extra mass, extra power extraction and extra drag which are caused by the ancillary equipment should be comprehensively considered, as well as the required quantity of fuel which is used to counteract these three extra items [50]. Without considering the weight of load HXes which is unavoidable in modern aircraft, the utilization of fuel as heat sink won’t cause any increasing of take-off weight, drag and power extraction, thus it will not bring any exergy penalty to the aircraft. Using SHX [51] and EHS as heat sink will increase the take-off weight of aircraft due to the mass of HX and expendable liquid, further leading to an exergy penalty. The utilization of RA [19] and EFA [45] as heat sinks will not only increase the take-off weight due to extra HXes, but also bring drag to the aircraft due to the momentum loss of cooling airflow, and both items will bring exergy penalty to the aircraft. Since there are great differences among heat sinks, different aircrafts require different designs of their specific TMS. Although the heat sinks mentioned above have attracted many researchers’ attention, the quantitative estimation of their cooling abilities/capacities and performance penalties under different flight conditions, as well as the comparison among heat sinks have never been systematically studied before.

This paper presents a numerical study about the cooling ability/capacity and exergy penalty of five kinds of heat sinks—fuel, RA, EFA, SHX, EHS—in different stages of a supersonic flight profile. In addition, the comparison of the cooling ability/capacity and exergy penalty of each heat sink is also presented in current study. In Section 2 of this paper, the structure and characteristic of various heat sinks as well as the methods they cool the thermal loads in the typical TMS have been introduced in detail. Section 3 presents several models for estimating the cooling ability/capacity of various heat sinks as well as the exergy penalty caused by these heat sinks, facilitate the quantification and comparison of various heat sinks. The exergy penalty presented in this paper takes into account the exergy loss of aircraft caused by the extra weight, drag and energy extraction of various heat sinks, but without considering the impact of the extra required fuel quantity caused by heat sinks. Section 4 elaborates a numerical case study in which the cooling ability/capacity and exergy penalty of each heat sink in different flight stages of a typical supersonic flight profile have been determined. The comparison and analysis also have been illustrated in this section. At last, based on the numerical study results, Section 5 presents the conclusions.

## 2. Heat Sinks of Modern Aircraft

**Figure 1:**
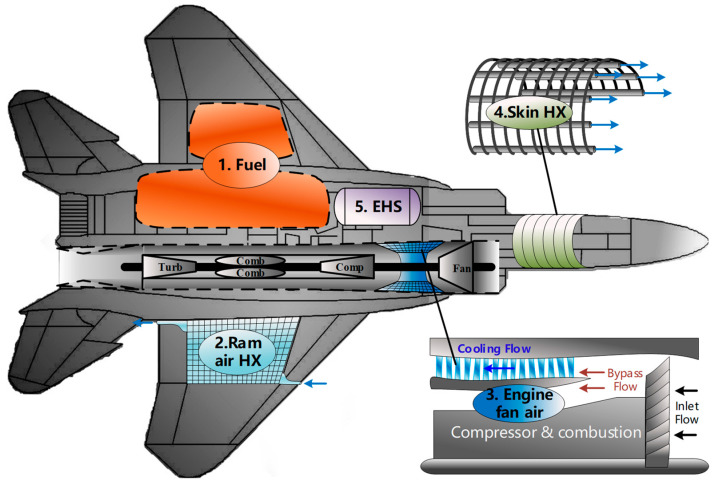
Typical heat sinks in modern aircraft. HX, heat exchangers; EHS, expendable heat sink.

This paper discusses five kinds of typical heat sinks of modern aircrafts: fuel, RA, EFA, SHX, and EHS. The distribution of each heat sink in an airborne vehicle is presented in Figure 1 which directly illustrates the locations of these heat sinks, as well as the basic configuration of each heat sink. First of all, fuel is the most important heat sink in most modern aircraft. It is stored in the fuel tanks around the fuselage and wings. Secondly, the RA is always introduced by protruding ram scoops or flush inlets, and then directly be sent to the equipment racks or separated cooling HXs to take away the heat from thermal loads. Thirdly, the EFA is bled from the external duct of the turbofan engine. There are two ways to utilize the fan air as a heat sink: (1) Duct the fan air to each separated mounted heat exchanger, or (2) go through the fan duct heat exchanger (FDHX) which is embedded inside the engine fan duct. The FDHX belongs to the annular radiator, which has the advantages of low air drag, invisibility, and good aerodynamic. Therefore, this study only considers the usage of the EFA through FDHX. Fourthly, the SHX rejects the heat of thermal loads directly to ambient through aircraft fuselage. There are generally three methods of implementing skin heat sink: (1) air loop, (2) liquid loop, and (3) heat pipe. This study mainly focuses the liquid loop because it has the advantages of strong heat transfer ability (compared with that of the air loop) and easy-to-control property (compared with that of the heat pipe). Using coolant as the heat transfer medium, the liquid loop transfer heat from thermal loads to the skin HX in which there is a series of tubes mounted between the metal plate and insulation layer of the skin which can directly dissipate heat to the ambient. At last, the EHS, which generally utilize the latent heat of liquid or endothermic chemical reaction to cool thermal loads, is stored in the special containers and will be vented to the atmosphere after vaporizing or exhausting. In this study, the liquid natural gas (LNG) and liquid ammonia (LA) are considered as EHS which will be discussed later.

**Figure 2:**
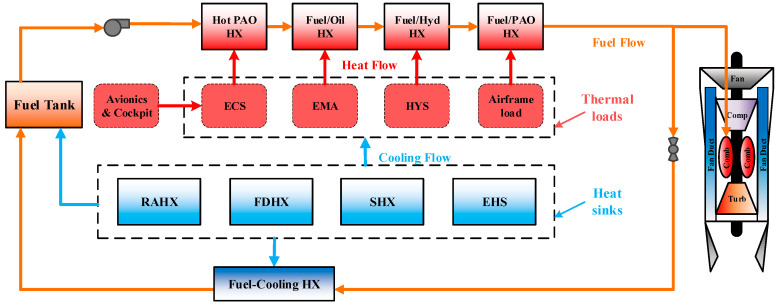
Typical thermal management system (TMS) of modern advantage aircraft. ECS, environment control system; EMA, electro-mechanical actuation; HYS, hydraulic system; RAHX, ram air heat exchanger; FDHX, fan duct heat exchanger; SHX, skin heat exchanger; PAO, Poly Alpha Olefin.

All kinds of heat sinks will realize the goal of cooling thermal loads through the TMS. Figure 2 illustrates the typical construction of the TMS of the modern aircraft. The typical TMS, shown in Figure 2, generally consists of a fuel tank or integrated tank system, several cooling HXs for various thermal loads of the environment control system (ECS), electro-mechanical actuation (EMA), and hydraulic system (HYS), and a fuel cooling circuit (marked by the origin line). The fuel cooling circuit can be further divided into two parts: the cooling channel that is upstream of the engine and the backflow channel that is behind the engine. Note that the ECS is a special thermal loads with complex structures which collects heat from avionics and cockpit through the vapor compression system, and then transports the heat to fuel or other heat sinks. In this paper, it can only be treated as a thermal load equipment. Besides, the red and blue line in Figure 2 refer to the heat flow, and cooling flow respectively. The red and blue square on the fuel flow channel indicate the load HXes (cooling thermal loads) and fuel-cooling HX (cooling reflux hot fuel), respectively.

In the TMS, the fuel is firstly pumped out of the tank and passes through a series of load HXes in the cooling channel to directly cool the thermal loads. After the temperature rises, the required fuel flow is fed into the engine for combustion, and the rest fuel flow will return to the tank through the reflux channel. A fuel-cooled heat exchanger may be installed in the reflux channel, which used to cool the reflux hot fuel. RAHX, FDHX, SHX, EHS can be used to directly cool the thermal loads, or cool the fuel. For example, the RAHX can be used for direct cooling of the ECS, and can also be used for cooling backflow hot fuel, for cooling fuel in the tank. The same with other heat sinks that are EFA, SHX, and EHS. The cooling ability and exergy penalty of various heat sinks in the TMS will be discussed below.

## 3. Estimation Models

The estimation models of cooling ability/capacity of fuel, RA, EFA, SHX, and EHS will be firstly developed in this section. The cooling ability whose unit is W is defined as the heat sink’s biggest heat transfer rate (HTR) for cooling the thermal loads, which is indicated by *q*. The cooling capacity whose unit is J is defined as the cooling quantity, which is indicated by *Q*. Secondly, the exergy penalties of different heat sinks will also be considered in this section. For conveniently comparing the costs when using different heat sinks, the exergy penalties of various heat sinks caused by extra mass, extra drag and power extracting, have also been determined, namely Ex. Note that all of the parameters in this paper are explained in the Nomenclature section, and some of the corresponding explanations will not be repeated.

### 3.1. Assumptions

Because the cooling ability and cooling capacity defined the largest HTR and cooling quantity of each heat sink, the ideal situation has been supposed in which the cooling capacity of each heat sink can be fully exploited. Several assumptions are presented as follows:

(1) Ignoring the heat leakage, all of the HS can be fully utilized.
(2) The efficiency of engine and lift-drag ratio of aircraft do not change during the whole flight period—this assumption is used to roughly estimate the fuel consumption and output power of engine.
(3) There is always enough heat load to be cooled by various heat sinks, and all the thermal loads are kept at the maximum allowable temperature *T_loax,_*_max_—this assumption provides an extreme condition for assessing the maximum cooling ability/capacity of various heat sinks, and it also provides the same work condition for different heat sinks for comparing their cooling ability/capacity.
(4) The heat transfer efficiency of each HX can be higher than a designed minimum value *η_HX,_*_min_ during the whole flight period—this assumption is used to determine the heat transfer efficiency of HXs under arbitrary conditions, as well as the additional weight caused by these HXs.

### 3.2. Fuel’s Cooling Ability and Residual Cooling Capacity

#### 3.2.1. Cooling Ability of the Fuel

Fuel’s cooling ability in this study refers to the HTR of fuel consumption flow rate of engine, which can be determined by Equation (1). Using the engine consumption fuel flow as heat sink can avoid the cooling capacity loss caused by hot fuel backflow. The cooling ability of fuel is mainly determined by its flow rate, temperature and threshold temperature. The threshold temperature of fuel is the maximum allowable temperature before it enters the engine:

(1) $$q_{fuel}=-{\dot{m}}_{fuel}c_{p,fuel}\left(T_{fuel,threshold}-T_{fuel}\right)$$

Firstly, according to assumption 2) above, the fuel mass flow $-{\dot{m}}_{fuel}$ that equals the engine fuel consumption can be determined by the engine efficiency and the current flight condition, shown as Equation (2). In this equation, *η_c_, η_m_* and *η_t_* refer to the combustion efficiency, mechanical efficiency and propulsive efficiency of the engine, respectively. *P_m,engine_* is the total mechanical power the engine supplies, while *P_t,engine_* is engine’s propulsive power, ${\dot{E}}_{k}$ indicates the kinetic energy change rate, ${\dot{E}}_{p}$ is potential energy change rate and ${\dot{E}}_{d}$ is the energy loss resulting from drag. According to the basic flight theory, ${\dot{E}}_{k}$, ${\dot{E}}_{p}$ and ${\dot{E}}_{d}$ all can be determined by the weight, flight velocity and altitude of the aircraft, shown as Equations (3)–(5) respectively. The weight of aircraft *m_a_* will decrease with the consumption of fuel, shown as Equation (6):

(2) $$-{\dot{m}}_{fuel}=\frac{P_{m,engine}}{\eta_{c}\eta_{m}\phi_{fuel}}=\frac{P_{t,engine}}{\eta_{c}\eta_{m}\eta_{t}\phi_{fuel}}=\frac{{\dot{E}}_{k}+{\dot{E}}_{p}+{\dot{E}}_{d}}{\eta_{c}\eta_{m}\eta_{t}\phi_{fuel}}$$

(3) $${\dot{E}}_{k}=d\left(m_{a}v_{a}^{2}/2\right)/dt=m_{a}v_{a}\cdot {\dot{v}}_{a}+v_{a}^{2}\cdot {\dot{m}}_{a}/2$$

(4) $${\dot{E}}_{p}=m_{a}g\cdot \dot{H}$$

(5) $${\dot{E}}_{d}=D_{a}\cdot v_{a}=m_{a}g\cdot v_{a}/K$$

(6) $${\dot{m}}_{a}={\dot{m}}_{fuel}$$

Secondly, without the hot fuel backflow, the temperature of the fuel mass in tank is determined by the HTR from tank to aircraft skin, shown as Equation (7) where $A_{tank}$ and $k_{tank}$ refer to the tank surface’s area and heat transfer coefficient from fuselage skin to tank, respectively. The skin temperature is considered to be equal to the total temperature of ambient air $T_{ex}$—also known as stagnation temperature—which can be determined by Equation (8), where the ambient temperature $T_{\infty }$ will be discussed in detail in next Section 3.3:

(7) $${\dot{T}}_{fuel}=\frac{A_{tank}k_{tank}\cdot \left(T_{ex}-T_{fuel}\right)}{m_{fuel}c_{p,fuel}}$$

(8) $$T_{ex}=T_{\infty }\left(1+0.2Ma^{2}\right)$$

#### 3.2.2. Residual Cooling Capacity of Fuel

On the other hand, the cooling capacity of the total fuel mass in tank can be estimated by the difference between fuel’s temperature and threshold temperature, as well as its remaining weight, shown by Equation (9). The cooling capacity of fuel will decrease with the reduced mass and the rising temperature:

(9) $$Q_{fuel}=m_{fuel}c_{p,fuel}\left(T_{fuel,threshold}-T_{fuel}\right)$$

#### 3.2.3. Exergy Penalty of Fuel

Different with most of other heat sinks, the using of engine consumption fuel flow will not cause any exergy penalties and even be advantageous to engine combustion efficiency. Thus the exergy penalty of fuel is equal to zero.

### 3.3. RA’s Cooling Ability and Exergy Penalty Rate

#### 3.3.1. Cooling Ability of RA

RA takes heat away from load coolant through the air-liquid HX which is known as RAHX. The cooling ability of RA refers to the maximum HTR from the heat loads to RA. According to the assumption 3) the temperature of the thermal load is always $T_{load,\max}$, the cooling ability of RA can be determined by Equation (10), where the cooling ability of RA $q_{RA}$ is directly determined by its mass flow rate ${\dot{m}}_{RA}$, total temperature $T_{ex,RA}$, and the heat transfer efficiency of RAHX *η_RAHX_*:

(10) $$q_{RA}=\max\left[{\dot{m}}_{RA}c_{p,air}\eta_{RAHX}\left(T_{load,\max}-T_{ex,RA}\right),0\right]$$

**Figure 3:**
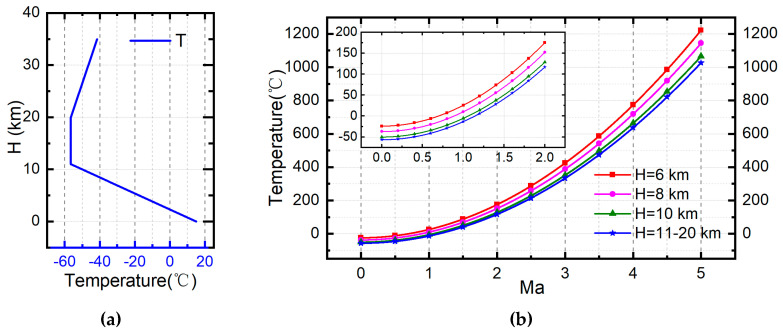
(**a**) Atmosphere temperature with altitude; (**b**) Total temperature of RA with flight Mach number.

Firstly, RA’s total temperature can be estimated by the ambient air temperature and flight Mach number. Suppose that the atmosphere parameters at sea level are as shown in Table 1, the ambient temperature, pressure and density varied with altitude in the troposphere (0 < h < 11 km) can be established by Equations (11)–(13) where γ=0.0065 K/m is the temperature lapse rate. Besides, the ambient temperature in the stratosphere (11 km < h < 20 km) will stay −56.5 °C and during the altitude of 20 km~32 km it will increase 1 K with elevation of sea level per 1 km. Since the Mach number of ram air is equal to the flight Mach number $Ma{\;}_{RA}=Ma$, the total temperature of RA can also be determined by Equation (8). The atmospheric temperature curve following with altitude is shown in Figure 3a, and the total temperature curve following with Mach number is shown in Figure 3b.

(11) $$T_{\infty }=T_{0}-\gamma H$$

(12) $$p_{\infty }=p_{0}{\left(1-\frac{H}{44330}\right)}^{\frac{g}{\gamma R}}$$

(13) $$\rho_{\infty }=\rho_{0}{\left(1-\frac{H}{44330}\right)}^{\frac{g}{\gamma R}-1}$$

Secondly, the RA’s mass flow rate ${\dot{m}}_{RA}$ can be estimated by its inlet area $A_{in,RA}$, inlet Mach number $Ma_{in,RA}$ and total pressure $p_{ex,RA}$, shown as Equation (14). In this equation, $Ma_{in,RA}$ is the Mach number at the inlet position of RA, considering the effect of shock wave under supersonic condition, which can be determined by Equation (15). Besides, the RA’s total pressure can be determined by Equation (16):

(14) $${\dot{m}}_{RA}=A_{in,RA}\cdot \frac{417Ma_{in,RA}}{{\left(1+0.2Ma_{in,RA}^{2}\right)}^{3.0}}\cdot \frac{p_{ex,RA}/p_{0}}{\sqrt{T_{ex,RA}/T_{0}}}$$

(15) $$Ma_{in,RA}=\left\{ \begin{array}{ll} Ma{\;}_{RA} & ,\;if:\;Ma_{RA}<1\\ \sqrt{\frac{Ma_{RA}^{2}+5}{7(Ma_{RA}^{2}-1)-1}} & ,if:\;Ma_{RA}\geq 1 \end{array}\right.$$

(16) $$p_{ex,RA}=p_{\infty }{\left(1+0.2Ma_{RA}^{2}\right)}^{3.0}$$

Finally, the heat transfer coefficient of RAHX only depends on its RA flow rate at the cold end, in the ideal situation, shown by Equation (17). In the equation, $k_{RAHX}$ and $A_{RAHX}$ represent its total heat transfer coefficient and effective heat transfer area, respectively. Their product $k_{RAHX}\cdot A_{RAHX}$ indicates the heat flux transferred through the HX when the average temperature difference is 1K. Since the efficiency $\eta_{RAHX}$ decreased with the increase of the RA flow rate ${\dot{m}}_{RA}$, the maximum RA flow rate will result in the minimum efficiency $\eta_{RAHX,\min}$ whose valve has been identified according to assumption 4). Therefore, the efficiency of RAHX under arbitrary conditions can be determined by contrasting the RA flow rate with its maximum value, shown in Equation (18):

(17) $$\eta_{RAHX}=1-\exp\left(-\frac{k_{RAHX}\cdot A_{RAHX}}{{\dot{m}}_{RA}c_{p,RA}}\right)$$

(18) $$\eta_{RAHX}=1-{\left(1-\eta_{RAHX,\min}\right)}^{\frac{{\dot{m}}_{RA,\max}}{{\dot{m}}_{RA}}}$$

Based on Equations (14) and (15), the RA flow rate will increase with increasing inlet Mach number $Ma_{in,RA}$, and the $Ma_{in,RA}$ will reach its maximum value when the RA’s Mach number $Ma_{RA}=1$. Therefore, the maximum RA flow rate can be approximately determined by Equation (19), where $T_{ex,RA,Ma=1}$ and $p_{ex,RA,Ma=1}$ are RA’s total temperature and total pressure respectively when Ma=1 at cruising altitude:

(19) $${\dot{m}}_{RA,\max}={\dot{m}}_{RA,Ma=1}=241.3\cdot A_{in,RA}\cdot \frac{p_{ex,RA,Ma=1}/p_{0}}{\sqrt{T_{ex,RA,Ma=1}/T_{0}}}$$

#### 3.3.2. Exergy Penalty Rate of Ra

The exergy penalty caused by using RA can be divided into drag penalty and the weight penalty of RAHX. Firstly, ignoring the negligible external drag—caused by friction, the resistance penalty of RA mainly comes from internal drag–caused by momentum loss of RA flow—which can be determined by Equation (20) where $\xi_{RA}$ is the velocity loss coefficient when RA passes through RAHX:

(20) $$D_{RA}={\dot{m}}_{RA}v_{RA}\cdot \xi_{RA}$$

Secondly, the structure of RAHX will lead to an increase in the take-off weight. HX’s weight can be determined by Equation (21) where the effective heat transfer area $A_{RAHX}$ can be determined by Equation (22) based on the Equation (17) mentioned above. It is noteworthy that this method for estimating the weight of HX is universal, and it is also applicable to the weight determination of FDHX and SHX later:

(21) $$m_{RAHX}=\rho_{RAHX}\cdot \frac{A_{RAHX}}{\alpha_{RAHX}}$$

(22) $$A_{RAHX}=-\frac{{\dot{m}}_{RA,\max}c_{p,RA}}{k_{RAHX}}\ln\left(1-\eta_{RAHX,\min}\right)$$

In order to compare the exergy penalties of different heat sinks more conveniently, the drag penalty and weight penalty caused by using RA should be quantified as an exergy penalty. At first the drag penalty directly results in the loss of engine thrust, so its exergy penalty rate can be determined by Equation (23). Secondly, the weight of RAHX essentially brings additional weight to the aircraft. Therefore, by using the same method as Equations (2)–(5), the exergy penalty rate caused by the weight of RAHX can be estimated by Equation (24), where the three items of the numerator are successive kinetic energy change rate, potential energy change rate and drag energy loss caused by the additional weight. At last, the total exergy penalty rate caused by using RA can be determined by Equation (25):

(23) $$\dot{E}x_{d,RA}=-D_{RA}\cdot v_{RA}/\eta_{t}$$

(24) $$\dot{E}x_{w,RA}=-\frac{m_{RAHX}v_{a}\cdot {\dot{v}}_{a}+m_{RAHX}g\cdot \dot{H}+m_{RAHX}g\cdot v_{a}/K}{\eta_{t}}$$

(25) $$\dot{E}x_{RA}=\dot{E}x_{d,RA}+\dot{E}x_{w,RA}$$

### 3.4. EFA’s Cooling Ability and Exergy Penalty Rate

#### 3.4.1. Cooling Ability of EFA

Similar to RA, EFA collects heat from thermal loads through the FDHX. The cooling ability of EFA can be estimated by Equation (26), which is influenced by the EFA’s mass flow rate ${\dot{m}}_{EFA}$, total temperature *T_ex,EFA_* and the heat transfer efficient of FDHX $\eta_{FDHX}$:

(26) $$q_{EFA}=\max\left[{\dot{m}}_{EFA}c_{p,air}\eta_{FDHX}\left(T_{load,\max}-T_{ex,EFA}\right),0\right]$$

Generally, the mass flow rate, total temperature of EFA and the heat transfer efficient of FDHX can be determined in the same way as RA. The only difference is that the EFA has been accelerated by the engine fan, so its Mach number *Ma_EFA_* will be bigger than that of RA, resulting in higher total temperature. Two important performance indexes of turbofan engine are bypass ratio *B* and power partition coefficient *X* who will directly influence the Mach number of EFA. The bypass ratio is the ratio between the mass flow rate of the bypass stream and the mass flow rate when entering the core. And the power partition coefficient is the proportion between available work that engine offers ducted fan and all mechanical work produced by engine. The power provided by the engine to per kilogram bypass stream $w_{b}$ can be determined by Equation (27) where $\eta_{f}$ is the efficiency of the duct fan which indicates the ratio of converting mechanical power into the kinetic energy of bypass stream. The $P_{m,engine}$ and ${\dot{m}}_{fuel}$ in these two equations have been obtained by Equation (2) above:

(27) $$w_{b}=\frac{P_{m,fan}}{{\dot{m}}_{core}}\cdot \eta_{f}=\frac{P_{m,engine}}{{\dot{m}}_{fuel}\cdot \theta }\cdot \frac{X}{1+B}\cdot \eta_{f}$$

Suppose that the static pressure of inlet and outlet of duct is equal, the Mach number of EFA—also is denoted as the Mach number of bypass stream in external duct which can be determined by Equation (28) where $\nu_{\infty ,sound}$ is the local sound speed. Then its total temperature $T_{ex,EFA}$, mass flow rate ${\dot{m}}_{EFA}$ and the efficient of FDHX $\eta_{FDHX}$ can be determined by using the same way as RA, shown as Equations (8), (14) and (18). The cooling ability can finally be determined by Equation (26):

(28) $$\left(Ma_{EFA}^{2}-Ma_{}^{2}\right)\cdot \nu_{\infty ,sound}=2w_{b}$$

#### 3.4.2. Exergy Penalty Rate of EFA

The exergy penalties of EFA can also be divided into drag penalty and weight penalty. Similar to RA, the drag caused by EFA and the weight of FDHX can be estimated by Equations (20) and (21). Then the exergy penalty rate caused by using EFA can be obtained according to Equations (29)–(31):

(29) $$\dot{E}x_{d,EFA}=-D_{EFA}\cdot v_{EFA}/\eta_{t}$$

(30) $$\dot{E}x_{w,EFA}=-\frac{m_{FDHX}v_{a}\cdot {\dot{v}}_{a}+m_{FDHX}g\cdot \dot{H}+m_{FDHX}g\cdot v_{a}/K}{\eta_{t}}$$

(31) $$\dot{E}x_{EFA}=\dot{E}x_{d,EFA}+\dot{E}x_{w,EFA}$$

### 3.5. SHX’s Cooling Ability and Exergy Penalty Rate

#### 3.5.1. Cooling Ability of SHX

The SHX is also a kind of ambient heat sink. Different from RA and EFA, its cooling ability is limited by the heat transfer of the available skin surface rather than that of the cooling air flow. The cooling ability of SHX can be estimated by Equation (32), where $T_{skin}$ is the average temperature of the skin which can be determined by the heat transfer efficient of SHX shown as Equation (33), and $k_{skin}$ is the heat transfer coefficient on the outside skin surface. The heat transfer efficiency of SHX $\eta_{SHX}$ is a design parameter in this part. Assuming that the outside skin is smooth and the boundary flow layer is laminar, the heat transfer coefficient can be estimated by Equation (34) where Re=ρv/μ and $\mathrm{Pr}=\mu c_{p}/\lambda$ are respectively the Reynolds number and Prandtl number:

(32) $$q_{SHX}=A_{skin}\left[k_{skin}\left(T_{skin}-T_{ex}\right)+\sigma \varepsilon_{skin}\left(T_{skin}^{4}-T_{\infty }^{4}\right)\right]$$

(33) $$T_{skin}-T_{ex}=\eta_{SHX}\left(T_{load,\max}-T_{ex}\right)$$

(34) kskin=0.332⋅λ∞⋅Re0.5⋅Pr13

#### 3.5.2. Exergy Penalty Rate of SHX

There is no drag penalty but weight penalty when using SHX as heat sink. Since only the hot end of SHX has the coolant as heat transfer medium, the efficiency of SHX is determined by the coolant flow rate. Similar with the Equation (20) and Equation (21), the weight of SHX can be determined by Equation (35), where the product ${\dot{m}}_{SHX,\max}c_{p,coolant}$ of SHX can be estimated by Equation (36). $q_{SHX,\max}$ is the maximum heat transfer capability of SHX which can be easily estimated. Therefore, the exergy penalty rate caused by SHX can be estimated by the weight penalty shown as Equation (37):

(35) $$m_{SHX}=-\rho_{SHX}\cdot \frac{{\dot{m}}_{SHX,\max}c_{p,coolant}\ln\left(1-\eta_{SHX}\right)}{\alpha_{SHX}k_{SHX}}$$

(36) $$q_{SHX,\max}={\dot{m}}_{SHX,\max}c_{p,coolant}\left(T_{load,\max}-T_{skin}\right)$$

(37) $$\dot{E}x_{SHX}=\dot{E}x_{w,SHX}=-\frac{m_{SHX}v_{a}\cdot {\dot{v}}_{a}+m_{SHX}g\cdot \dot{H}+m_{SHX}g\cdot v_{a}/K}{\eta_{t}}$$

### 3.6. EHS’s Cooling Capacity and Exergy Penalty Rate

The cooling ability of EHS is meaningless because the HTR of EHS can be easily modified according to demand. Thus in this part author only discusses the cooling capacity of EHS, as well as its penalty.

#### 3.6.1. Cooling Capacity of EHS

The EHS cools the thermal loads mainly depending on the heat absorption when changing phase. Under an ideal condition, the EHS after being used can expand to the ambient pressure and rise to the temperature equal to the heat load, thus its cooling capacity can be determined by Equation (38), where the $h_{liquid}$ is the special enthalpy of EHS before phase change and $h_{gas}$ is the special enthalpy after phase change. $T_{st,EHS}$ is the storage temperature of EHS, and $p_{st,EHS}$ is the storage pressure which is considered as the saturation pressure of the expendable liquid under the storage temperature in this study:

(38) $$Q_{EHS}=m_{EHS}\cdot \left[h_{gas,EHS}(T_{load,\max},p_{\infty })-h_{liquid,EHS}(T_{st,EHS},p_{st,EHS})\right]$$

#### 3.6.2. Exergy Penalty Rate of EHS

The penalty caused by EHS belongs to weight penalty. The additional weight caused by EHS includes its own weight and the container’s weight. Generally the container’s weight is in proportion to the weight of EHS and the ratio is φ. Therefore, the exergy penalty rate of EHS can be evaluated by Equation (39):

(39) $$\dot{E}x_{EHS}=\dot{E}x_{w,EHS}=-(1+\varphi )m_{EHS}\cdot \frac{v_{a}\cdot {\dot{v}}_{a}+g\cdot \dot{H}+g\cdot v_{a}/K}{\eta_{t}}$$

### 3.7. Cooling Capacity and Exergy Penalty of Heat Sinks

Section 3.2, Section 3.3, Section 3.4, Section 3.5 and Section 3.6 illustrated the estimation models for the cooling ability of fuel, RA, EFA, SHX, and the exergy penalty rate of RA, EFA, SHX, EHS. On this basis, the cooling capacity and exergy penalty of each heat sink during a target flight period can be determined by integrating the cooling ability and exergy penalty rate in this time range $t_{1}\to t_{2}$, as shown in Equations (40) and (41) respectively. For evaluating the advantages and disadvantages of each heat sink more conveniently, the parameter cooling-penalty ratio has also been introduced as Equation (42). The cooling-penalty ratio is the ratio of cooling capacity and exergy penalty. The higher the value, the more cooling quantity the heat sink can offer when consuming unit energy, further indicating that it is more ideal as a heat sink:

(40) $$Q^{\left(t_{1}\to t_{2}\right)}=\int_{t_{1}}^{t_{2}}q^{\left(t\right)}dt$$

(41) $$Ex^{\left(t_{1}\to t_{2}\right)}=\int_{t_{1}}^{t_{2}}\dot{E}x^{\left(t\right)}dt$$

(42) $$\chi^{\left(t_{1}\to t_{2}\right)}=\left|\frac{Q^{\left(t_{1}\to t_{2}\right)}}{Ex^{\left(t_{1}\to t_{2}\right)}}\right|$$

### 3.8. Calculation Procedure

**Figure 4:**
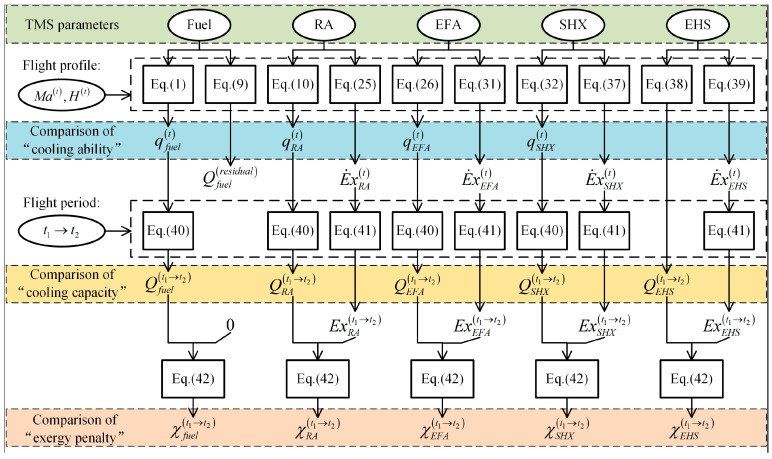
The calculation procedures.

Basically, there are two main purposes to be discussed in this study. The first one is the influence of various design parameters of TMS on the cooling ability/capacity and exergy penalty of each heat sink, which can be used to guide the design and optimization of various heat sink. The second one is the comparison of different heat sinks, which can be used to more reasonably arrange the proportion of various heat sinks according to target flight mission. For these two purposes, calculation procedures are illustrated in Figure 4.

As shown in Figure 4, the square and circular frames in the figure indicate the corresponding equations and parameters respectively. The calculation procedures include three steps. Firstly, based on the parameters of each heat sink and the flight profile, the cooling ability of fuel, RA, EFA and SHX at any time can be determined by corresponding equations, i.e., $q_{fuel}^{\left(t\right)}$, $q_{RA}^{\left(t\right)}$, $q_{EFA}^{\left(t\right)}$, $q_{SHX}^{\left(t\right)}$. By the same way, the exergy penalty rates of various heat sinks can be obtained, i.e., $\dot{E}x_{RA}^{\left(t\right)}$, $\dot{E}x_{EFA}^{\left(t\right)}$, $\dot{E}x_{SHX}^{\left(t\right)}$, $\dot{E}x_{EHS}^{\left(t\right)}$. Note that the cooling ability of EHS is meaningless and the exergy penalty of fuel is 0. Secondly, based on the cooling ability and exergy penalty rate of each heat sink obtained in the first step, the total cooling capacity of each heat sink during a target flight stage $t_{1}\to t_{2}$ can be determined by corresponding Equation (40), i.e., $Q_{fuel}^{\left(t_{1}\to t_{2}\right)}$, $Q_{RA}^{\left(t_{1}\to t_{2}\right)}$, $Q_{EFA}^{\left(t_{1}\to t_{2}\right)}$, $Q_{SHX}^{\left(t_{1}\to t_{2}\right)}$, $Q_{EHS}^{\left(t_{1}\to t_{2}\right)}$. By the same way, the exergy penalty of each heat sink can be determined by Equation (41), i.e., 0, $Ex_{RA}^{\left(t_{1}\to t_{2}\right)}$, $Ex_{EFA}^{\left(t_{1}\to t_{2}\right)}$, $Ex_{SHX}^{\left(t_{1}\to t_{2}\right)}$, $Ex_{EHS}^{\left(t_{1}\to t_{2}\right)}$. Thirdly, based on the cooling capacity and exergy penalty data obtained in step 2, the cooling-penalty ratio of each heat sink in target flight stage can be determined, i.e., $\chi_{fuel}^{\left(t_{1}\to t_{2}\right)}$, $\chi_{RA}^{\left(t_{1}\to t_{2}\right)}$, $\chi_{EFA}^{\left(t_{1}\to t_{2}\right)}$, $\chi_{SHX}^{\left(t_{1}\to t_{2}\right)}$, $\chi_{EHS}^{\left(t_{1}\to t_{2}\right)}$.

Based on the calculation results, the two purposes will be described at the beginning of this part. First, the influence of a target TMS parameter on one heat sink can be evaluated by the calculation results under different cases with different target parameters. Secondly, the comparison of different heat sinks can be further divided into three parts. (1) The comparison of each heat sink’s cooling ability can be carried out by comparing $q_{fuel}^{\left(t\right)}$, $q_{RA}^{\left(t\right)}$, $q_{EFA}^{\left(t\right)}$, $q_{SHX}^{\left(t\right)}$ under different flight conditions. (2) The comparison of each heat sink’s cooling capacity can be carried out by comparing $Q_{fuel}^{\left(t_{1}\to t_{2}\right)}$, $Q_{RA}^{\left(t_{1}\to t_{2}\right)}$, $Q_{EFA}^{\left(t_{1}\to t_{2}\right)}$, $Q_{SHX}^{\left(t_{1}\to t_{2}\right)}$ and $Q_{EHS}^{\left(t_{1}\to t_{2}\right)}$ during different flight stages; 3) the comparison of each heat sink’s exergy penalty can be carried out by comparing $\chi_{fuel}^{\left(t_{1}\to t_{2}\right)}$, $\chi_{RA}^{\left(t_{1}\to t_{2}\right)}$, $\chi_{EFA}^{\left(t_{1}\to t_{2}\right)}$, $\chi_{SHX}^{\left(t_{1}\to t_{2}\right)}$ and $\chi_{EHS}^{\left(t_{1}\to t_{2}\right)}$ during different flight stages. Please note that it is meaningless to directly compare each heat sink’s exergy penalty, since their cooling capacities are obviously different.

## 4. Results and Discussion

This section will mainly describe the application of the present model in hypothetical cases, aiming to verify the models mentioned above and discuss the estimating results. The calculation process was carried out by the software MATLAB/Simulink R2016a (MathWorks, Natick, MA, USA) in this study. Besides, the thermo-physical parameters of RA, EFA and EHS at any time, such as their thermal conductivity, special heat and special enthalpy. are those determined by the National Institute of Standards and Technology (NIST: Gaithersburg, MD, USA) in this study.

### 4.1. Case Design

This part will present a standard case “Case 0”, for estimating the cooling ability and cooling capacity of each heat sink under typical flight conditions. The case design mainly includes two steps. The first one is the determination of the basic parameters used in the “estimating model” presented in Section 3, and the second one is the design of a common flight profile which means the value of flight Mach number and altitude during the whole flight period. The rules for determining these parameters are based on the average level of TMS of the existing advanced aircrafts. When the exact value of a parameter is hardly confirmed, a reasonable value according to the common sense of aircraft and heat transfer theory will be applied, such as the detailed data of the FDHX and EHS.

#### 4.1.1. The Standard “Case 0” for Estimating Study

The basic parameters for estimating the cooling ability of various heat sinks in current case study are as listed in Table 2, which mainly includes the performance parameters of the aircraft and the design parameters of each heat sink. The performance parameters of aircraft, which can directly influence the fuel consumption rate and the Mach number of EFA, including the take-off weight of aircraft, the efficiency of engine, the lift-drag ratio and so on. The design parameters of heat sinks mainly include the initial fuel mass, initial fuel temperature, tank surface area, special heat and combustion value of fuel, the substance of EHS, and initial EHS mass. Besides, the structure parameters of RAHX, FDHX, SHX as well as the inlet area of RA and EFA are also the key to estimating the cooling capability of RA, EFA and SHX, which have been elaborated in Table 3. Please note that, the values in Table 3 are determined according to average level of the plate-fin HXs used in aviation. 

#### 4.1.2. Flight Profile

**Figure 5:**
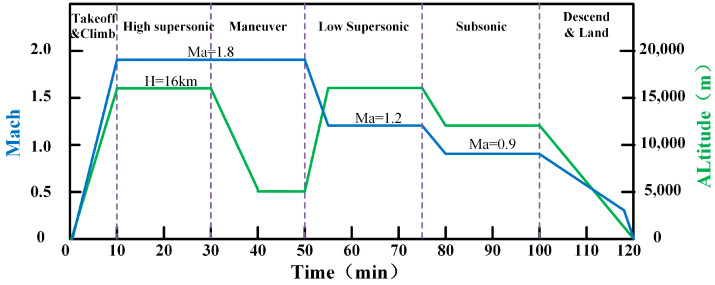
A typical mission profile of modern supersonic aircraft.

The second part of the case design is to determine a typical flight profile, which can obviously influence the cooling ability of each heat sink. For example, bigger engine fuel consumption rate can result in greater cooling ability of fuel flow, and faster flight Mach number will lead to higher total temperature of RA and EFA which will reduce their cooling ability. For more conveniently estimating the CAB of various heat sinks under different flight stages in advanced aircraft, an aircraft with the capabilities of supersonic, low supersonic and subsonic cruise is proposed, and a two-hour mission profile shown in Figure 5 is elaborated. 

The flight profile is divided into five typical flight stages. In the figure, the symbols ①~⑥ refer to the flight stages 1~6, respectively, the same as in the figures that follow. Stage 1: take off and climb (0~10 min). Stage 2: supersonic cruise at Mach 1.8 and at the altitude of 16,000 m (10~30 min). Stage 3: maneuver stage (30~50 min). Stage 4: low supersonic cruise at Mach 1.8 and at the altitude of 16000m (50~75 min). Stage 5: subsonic cruise at Mach 0.9 and at the altitude of 12,000m (75~100 min). Stage 6: descend and land (100~120 min). The detail date of flight Mach number and flight altitude during whole flight mission can be extract from the flight profile.

### 4.2. Influence of TMS Parameters on Heat Sinks

In this part we mainly investigate the influence of various design parameters of TMS on the cooling ability/capacity and exergy penalty of each heat sink. The estimation results of each heat sink during the whole flight mission will be determined for the different cases in this part. Each case is determined by changing one target parameter on the basis of “Case 0”. The change rules of each heat sink’s cooling ability/capacity and exergy penalty with this target parameter can be obtained from the calculation results. The target changing parameters mainly include the RA inlet area, FDHX frontal area, the substance and initial mass of EHS, and the efficiency of SHX. 

#### 4.2.1. Fuel

There are two results for simulating fuel as heat sink: the cooling ability of consumption fuel flow and the cooling capacity of remainder fuel mass in tank. Firstly, based on the performance parameters of the aircraft that is the flight Mach number and altitude, the heat transferred from ambient to tank and the fuel consumption rate during whole flight profile can be estimated.

**Figure 6:**
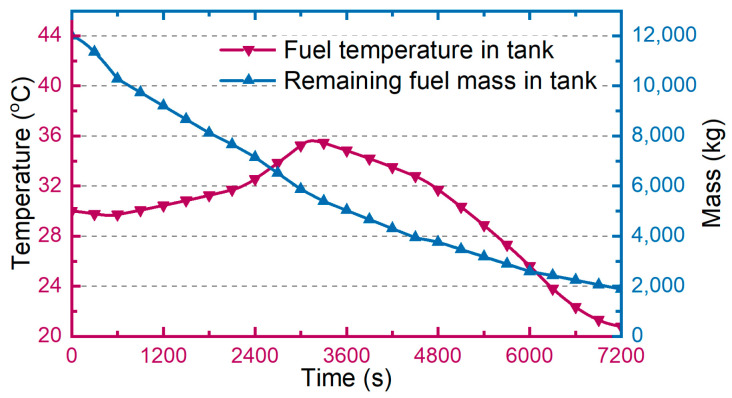
The remaining mass and temperature of fuel in tank during whole flight profile in Case 0.

Secondly, the remaining mass and temperature of fuel in tank can be easily determined by the conservation of mass and energy, and they are shown in Figure 6. In this study the hot fuel backflow is not considered, so the fuel temperature in actual case with backflow is usually higher, which leads to the decrease of the cooling ability and cooling capacity.

**Figure 7:**
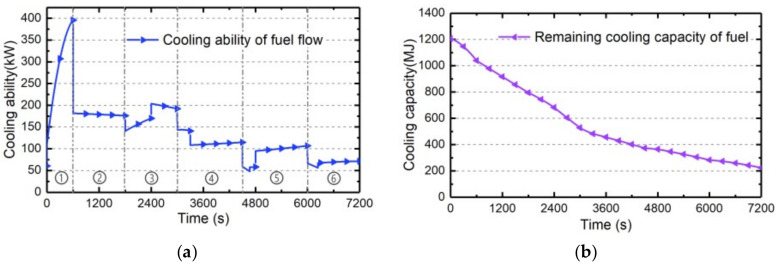
The estimation results of fuel in Case 0. (**a**) cooling ability of consumption fuel flow; (**b**) cooling capacity of remaining fuel mass in tank.

Finally, the cooling ability of consumption fuel flow and the cooling capacity of remaining fuel mass in tank can be determined by Equation (9), shown in Figure 7

#### 4.2.2. Ram Air

The cooling ability of RA and its exergy penalty rate with different ram inlet sizes are investigated in this part. The ram inlet area will directly determine the mass flow rate of RA, further influencing the cooling ability and exergy penalty rate of RA. On the basis of the standard case “Case 0” presented in Section 4.1, the Cases 1~5 with different ram inlet sizes are established as Table 4. The estimating results under these five cases are as follows. 

**Figure 8:**
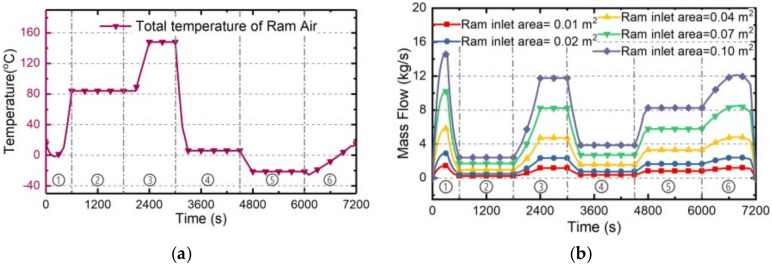
The estimating results of RA in Cases 1~5: (**a**) The total temperature and (**b**) The mass flow rate.

Firstly, the total temperature curve and mass flow rate under Cases 1~5 during the whole flight profile can be calculated by Equation (8) and Equation (14), respectively, as shown in Figure 8a and Figure 5b. Since the total temperature of RA is only determined by the flight Mach number and altitude, it is not affected by the ram inlet size. On the other hand, the mass flow rate of RA obviously increases with the increasing inlet area.

**Figure 9:**
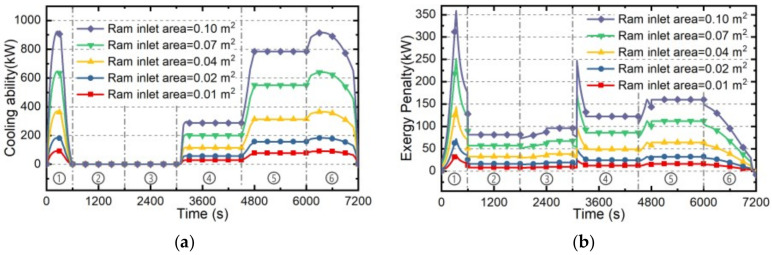

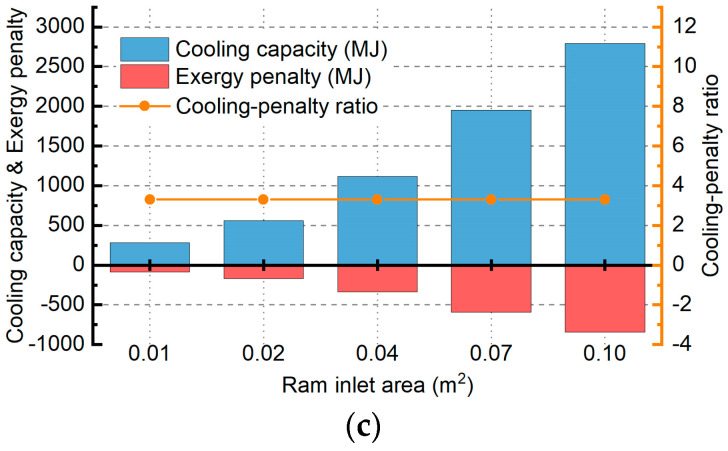
The estimation results of RA under Cases 1~5. (**a**) cooling ability; (**b**) exergy penalty rate; (**c**) cooling capacity and exergy penalty.

Secondly, the cooling ability of RA can be estimated by Equation (10), and the corresponding penalty rate can be calculated by Equation (25). The estimation results in Cases 1~5 are shown in Figure 9a,b, respectively. The cooling ability of RA increases with the increase of mass flow rate and is inversely proportional to its total temperature. Since the mass flow rate is directly proportional to the ram inlet area, both the cooling ability and exergy penalty rate of RA increase with the increasing ram inlet size. Note that when total temperature beyond the maximum load temperature meaning the cooling ability drops to zero, the mass flow rate will cut to zero to reduce the drag penalty of RA.

Thirdly, based on the cooling ability and exergy penalty rate of RA under Cases 1~5, the corresponding total cooling capacities and exergy penalties during whole flight mission can be determined by Equations (40) and (41), respectively, shown in Figure 9c. The cooling capacity and exergy penalty will all increase with the increase of ram inlet area. Besides, the cooling penalty ratio determined by Equation (42) which will only vary with flight condition, is constant (3.31 during whole flight mission) when the ram inlet size is changing.

#### 4.2.3. Engine Fan Air

The cooling ability of EFA and the exergy penalty rate during whole flight profile have been estimated with different FDHX frontal sizes. Similar with the ram inlet area of RA, the frontal size of FDHX directly determines the mass flow rate of EFA, further influencing its cooling ability and exergy penalty rate. On the basis of the standard case “Case 0” presented in Section 4.1, the Cases 6~10 with different FDHX frontal area have been established in Table 5. The estimating results under Cases 6~10 are as follows.

**Figure 10:**
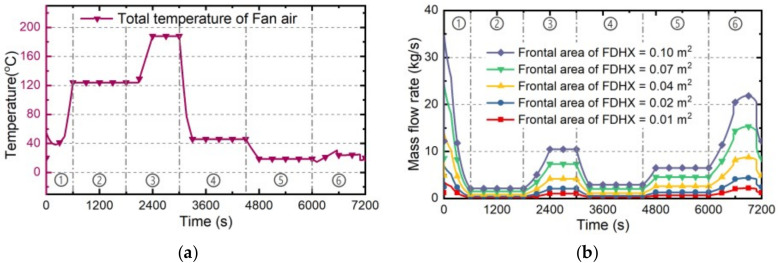
The estimating results of EFA in Cases 6~10. (**a**) total temperature; (**b**) mass flow rate.

Firstly, the total temperature curve of EFA doesn’t change with the frontal size, which is shown as Figure 10a. Since the EFA has been accelerated by the engine duct fan, its total temperature is higher than the RA. The mass flow rate curves of EFA under Cases 6~19 are shown as Figure 10b, which are obviously proportional to the frontal area of FDHX.

**Figure 11:**
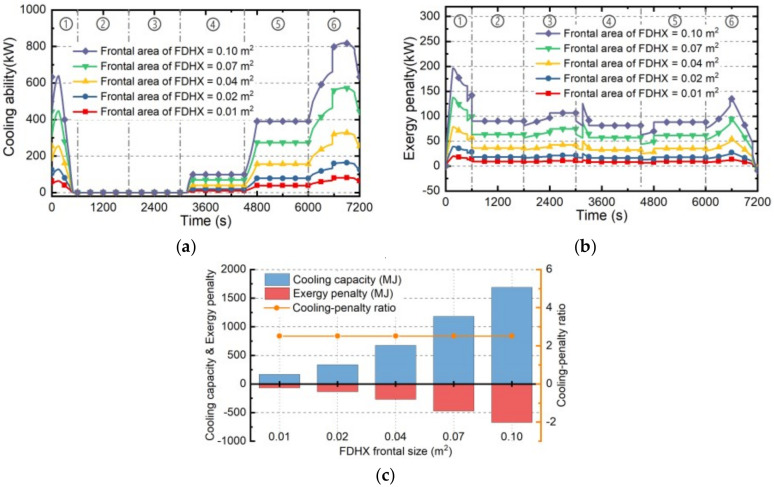
The estimation results of EFA under Cases 6~10: (**a**) cooling ability; (**b**) exergy penalty rate; (**c**) cooling capacity and exergy penalty.

Secondly, the cooling ability and the exergy penalty rate of EFA can be determined by Equation (26) and Equation (31). The corresponding estimation results under Cases 6~10 are shown as Figure 11a,b. Generally, the cooling ability of EFA is proportional to its total temperature inversely and proportional to the mass flow rate directly. On the other hand, the larger frontal area of FDHX can lead to higher mass flow rate, further bringing greater exergy penalty rate.

Thirdly, based on the cooling abilities and exergy penalty rates of EFA under Cases 6~10, the corresponding cooling capacities and exergy penalties of EFA during whole flight mission can be determined by Equations (40) and (41) respectively, shown in Figure 11c. Similar with RA, the cooling capacity and exergy penalty of EFA will all increase with the increasing FDHX frontal size. Besides, EFA’s cooling penalty ratio is constant (2.51 during whole flight mission) when the FDHX frontal size is changing.

#### 4.2.4. Skin HXs

The cooling ability and the exergy penalty rate of SHX under different heat transfer efficiency are presented in this part. The efficiency of SHX is actually determined by the scale of the HX, larger scale will lead to more hot coolant on the hot end of SHX, further bring higher temperature on the skin surface and improving the heat transfer efficiency. Therefore, higher heat transfer efficiency means stronger cooling ability and greater weight of SHX. On the basis of the standard case “Case 0” presented in Section 4.1, Cases 11~15 have been established as shown in Table 6. The estimating results of Cases 11~15 are as follows.

**Figure 12:**
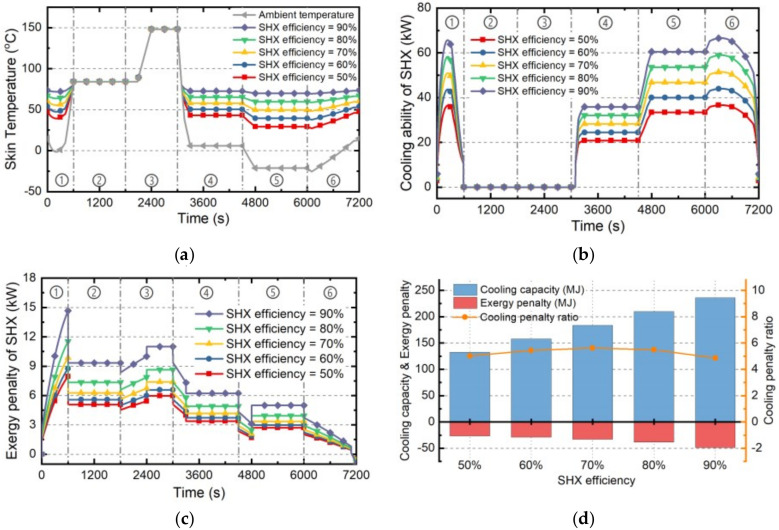
The estimation results of SHX under Cases 11~15. (**a**) average skin temperature; (**b**) cooling ability; (**c**) exergy penalty rate; (**d**) cooling capacity and exergy penalty.

Firstly, the sink temperature which can be determined by Equation (33) is the index of the balance between ambient cooling and internal heat transfer of SHX. The skin temperature curves under Cases 11~15 are illustrated as Figure 12a. Generally, it will increase with the increase of SHX efficiency and vary with the ambient temperature.

Secondly, the cooling ability and exergy penalty rate of SHX can be estimated by Equations (32) and (37), corresponding results under Cases 11~15 are shown as Figure 12b,c. Generally, the cooling ability of SHX which is inversely proportional to the skin temperature will increase with the increase of SHX efficiency. On the other hand, higher efficiency will result in greater SHX weight, further causing higher exergy penalty rate.

Thirdly, based on the cooling abilities and exergy penalty rates, the corresponding cooling capacities and exergy penalties of SHX during whole flight mission can be determined by Equations (40) and (41), shown in Figure 12d. Besides, the cooling-penalty ratio can be determined by Equation (42). The cooling capacity and exergy penalty of SHX during whole flight mission will increase with the increase of SHX’s efficiency while the cooling-penalty ratio will change slightly and achieve maximum when SHX’s efficiency is 70%.

#### 4.2.5. Expendable Heat Sinks

In this part we will discuss the cooling capacity and exergy penalty of EHS in different substances and different masses. Different substances will absorb different heats when changing phase, resulting in different cooling capacity. The initial mass directly determines the cooling capacity of EHS and also influences the exergy penalty. On the basis of the standard case “Case 0” presented in Section 4.1, Table 7 elaborates Cases 16~25 in which LA and LNG with diverse initial mass have been chosen as EHS.

**Figure 13:**
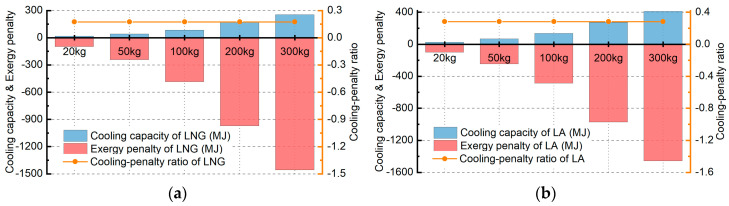
The cooling capacity and exergy penalty of EHS: (**a**) liquid natural gas; (**b**) liquid ammonia.

No matter what substance EHS is, it uses the heat absorption when phase changing to cool the thermal loads, and its penalty is only determined by the weight. The cooling capacity and corresponding exergy penalty of EHS (LNG or LA) can be determined by Equations (38) and (39), shown as Figure 13, in addition, the cooling-penalty ratio can be determined by Equation (42). Compared with the above four heat sinks, the cooling capacity of EHS is very limited and its energy penalty is too large, making it seems uneconomical. However, considering that the EHS can be used in cryogenic cooling, it is still valuable. LA’s cooling capacity is a little bigger than that of LNG under the same mass since it owns higher latent heat of evaporation. Besides, the LA can be stored at ambient temperature to make it safer than LNG. However, it should be noted that the LNG can be used for afterburner of the engine or generating power for the secondary energy system. 1 kilogram LNG can release heat of 46 MJ when burned. If the LNG could be fully utilized, according to the energy usage ratio of fuel, it can provide 14.5 MJ mechanical energy per kilogram for the aircraft which is quite considerable. Therefore, if the technical problems can be solved in the future, the combustible expendable liquid is the best choice for EHS.

### 4.3. Comparison of Different Heat Sinks

In this part all kinds of heat sinks will be compared on the basis of three aspects. All the analysis is based on the calculation results of “Case 0”. The first one is the cooling ability difference of various heat sinks under each flight condition. The cooling ability of heat sinks are quite different from each other and obviously vary under different flight conditions. The comparison of the cooling ability between various heat sinks can guide the strategy of using heat sink at different flight stages. Secondly, the cooling capacities and exergy penalties of various heat sinks are compared with each other during each flight stage as well as whole flight profile. At the same flight stage, the cooling capacities of different heat sinks are different, as well as their exergy penalties. The cooling capacity and exergy penalty of the same heat sink will obviously vary at different stage. The cooling capacity and exergy penalty of one heat sink at one flight stage directly reflects the effectiveness during this period—higher cooling capacity and lower exergy penalty are beneficial to the TMS and even entire aircraft. The comparison of cooling capacity of each heat sink at different flight stages can be used to select more suitable heat sink according to the flight mission and to adjust the ratios of various heat sinks more appropriately.

#### 4.3.1. Cooling Ability Comparison of Each Heat Sink

**Figure 14:**
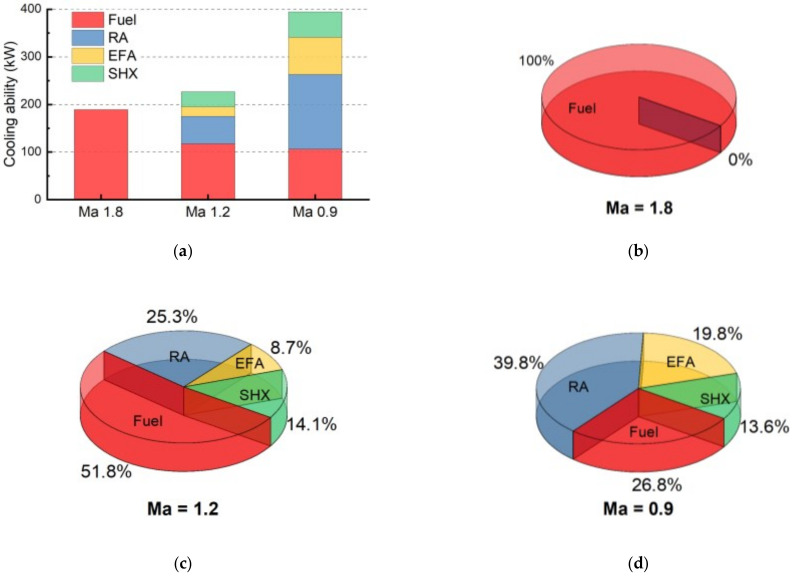
The comparison of cooling ability of various heat sinks at different flight stages. (**a**) overview; (**b**) supersonic at Ma 1.8; (**c**) low supersonic at Ma 1.2; (**d**) subsonic at Ma 0.9.

In this part the authors compare the cooling ability of each heat sink at different flight stages. Since the cooling ability of EHS is meaningless, only the cooling ability of fuel, RA, EFA and SHX in three cruise phases—supersonic at Ma 1.8, low supersonic at Ma 1.2, subsonic at Ma 0.9—are considered. The cooling ability of each heat sink at different flight Mach number is elaborated in Table 8, in which the values are extracted from 20 min, 65 min and 90 min of the estimating result of “Case 0” respectively. Besides, based on these values, the comparison can be illustrated in Figure 14.

As shown in Figure 14a, the cooling ability of fuel decreases with the decrease of flight Mach number, while that of RA, EFA and SHX all increase. Together, the total cooling ability will increase with the decreasing Mach number. On the other hand, the proportion of fuel’s cooling ability is gradually decreasing in the total cooling ability with decreasing Mach number. As shown in Figure 14b–d, the proportions of fuel’s cooling ability are 100%, 51.8% and 26.8%, when the Mach numbers are 1.8, 1.2 and 0.9 respectively. Under the flight condition of Ma 1.8, only fuel has cooling ability, while other heat sinks—the ambient heat sinks—have no cooling ability. The proportions of the cooling ability of RA, EFA and SHX are increase with the decrease of Mach number. 

#### 4.3.2. Cooling Capacity and Exergy Penalty Comparison of Each Heat Sink

**Figure 15:**
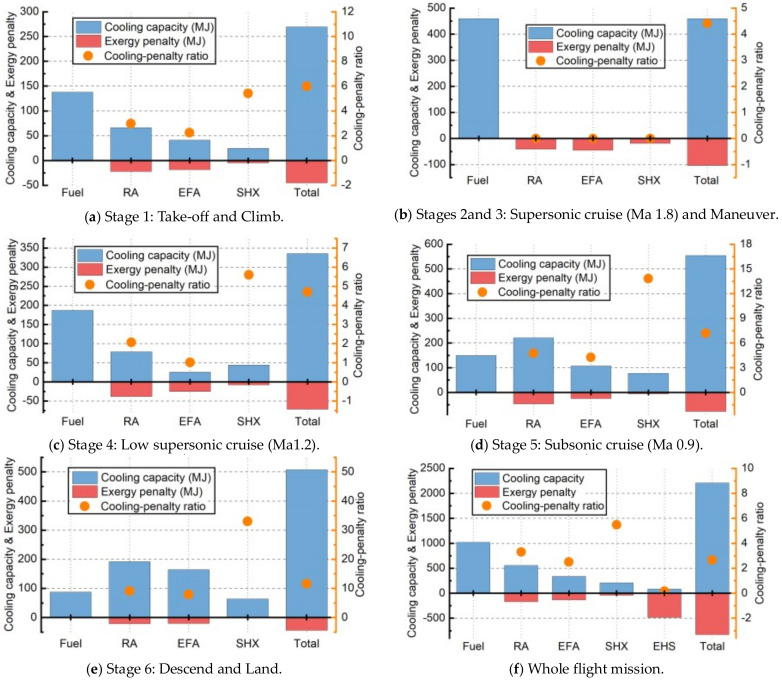
The comparison of cooling capacity of various heat sinks in different flight stages. (**a**) stage 1: take-off and climb; (**b**) stages 2 and 3: supersonic cruise (Ma 1.8) and maneuver; (**c**) stage 4: low supersonic cruise (Ma1.2); (**d**) stage 5: subsonic cruise (Ma 0.9); (**e**) stage 6: descend and land; (**f**) whole flight mission.

According to Equations (40) and (41) on the basis of the “Case 0”, the cooling capacities and exergy penalties of fuel, RA, EFA, SHX and EHS in various flight stages and whole flight mission have been determined as Figure 15, when the cooling-penalty ratios are determined by Equation (42). Besides, the corresponding cooling-penalty ratios determined by Equation (42) are also elaborated in Figure 15. There are three points that should be noted. Firstly, since the characteristics of each heat sink’s cooling capacity and exergy penalty in stage 3 (maneuver stage) are similar to those in stage 2 (supersonic cruise), the data of these two stages are discussed together in this study. Secondly, only EHS’s cooling capacity during whole flight mission has been considered, because its cooling capacity can be allocated arbitrarily without any restrictions. Thirdly, the duration time of stage 1, 2 and 3, 4, 5, 6 are not the same, namely 10, 40, 25, 25 and 20 min. Therefore, it is meaningless to compare the total cooling capacity and exergy penalty of each stage. 

As shown in Figure 15, fuel has the greatest cooling capacity in the whole flight mission, followed by RA, EFA, SHX and EHS, and the rank of exergy penalty from small to large is EHS, RA, EFA, SHX and fuel. On the other hand, comparing the cooling-penalty ratio values, their rank can be $\chi_{fuel}$>$\chi_{SHX}$>$\chi_{RA}$>$\chi_{EFA}$>$\chi_{EHS}$ during the whole flight mission. Note that the cooling-penalty ratio can only evaluate the heat sinks from the perspective of thermal management and without considering other factors. Therefore, some advantages of heat sinks are not considered. The detailed characteristics of each heat sink are discussed in follow.

Firstly, the cooling capacity of fuel is sufficient at any flight stage and is the largest during the whole flight mission. At the same time, fuel doesn’t cause any exergy penalty, resulting in the biggest cooling-penalty ratio (∞) and making fuel the most ideal heat sink. The only disadvantage is that its cooling capacity is limited by its storage mass in tank and will decrease seriously with the consumption of fuel in the later period of flight mission.

Secondly, the cooling capacity of RA is considerable at low Mach number, even exceeding the fuel. Besides, the cooling-penalty ratios of RA in stage 1, 2&3, 4, 5, 6 are 2.97, 0, 2.06, 4.74 and 9.07 respectively, which basically increase with the decrease of flight Mach number and flight altitude. This is because lower flight Mach number will lead to lower total temperature and higher mass, and at the same time, the lower flight Mach number causes lower exergy penalty. 

Thirdly, the situation of EFA is similar to that of RA. Although the cooling capacity of EFA is slightly lower than that of RA due to the higher total temperature, its exergy penalty of EFA is also lower than that of RA due to the advantage of the structure of FDHX. The FDHX belongs to the annular radiator which has lower drag penalty than traditional plate-fin HXs under the same heat transfer ability. The cooling-penalty ratio of EFA is a little lower than RA, which is worse than RA as the heat sink from the perspective of thermal management. But from the perspective of modern advanced aircraft designing, it has several advantages that RA cannot replace. (1) FDHX is more conducive to the stealth and aerodynamic design of aircraft. (2) EFA can also be used in standby state in airport as long as the engine is working, which makes the pre-cooling of fuel possible.

Fourthly, since the SHX integrates aircraft skin to reduce the weight and has no drag penalty, it has a high cooling-penalty ratio and is more ideal than the other two ambient heat sinks. However, its cooling capacity is strongly limited by the effective heat transfer area of the skin, which is much smaller than that of RA and EFA. Therefore, its function rapidly gets weakened on modern advantage aircrafts with higher and higher thermal load.

At last, the EHS owns a very little cooling-penalty ratio (0.17) which indicates that the EHS seems do not to be suitable as a heat sink. However, this is only considered from the perspective of thermal management. It still has two unique advantages: (1) EHS can provide cryogenic cooling for some special equipment who need a low temperature working condition such as laser diode. (2) If choosing a combustible substance such as LNG, it can be used for engine afterburning or power generation for a secondary energy system. Therefore, EHS has great potential in the future when the corresponding technical difficulties have been overcome.

## 5. Conclusions

In order to facilitate the design and optimization of the TMS for the increasingly severe thermal problems of modern advanced aircraft, this paper presents various models for estimating the cooling abilities/capacities of heat sinks and the exergy penalty caused by each heat sink. The target five heat sinks include fuel, RA, EFA, SHX and EHS. A heat sink is more desirable when its cooling ability/capacity is stronger while the exergy penalty is smaller. The cooling ability/capacity of each heat sink is not only related to its design parameters and thermo-physical states, but also varies with the flight condition of aircraft. Therefore, the TMS in different types of aircrafts should be obviously different.

The analysis results and discussion have been elaborated in this paper, that is the influences of design parameters on cooling ability/capacity and exergy penalty of various heat sinks, and the comparison of different heat sinks on the cooling ability/capacity and exergy penalty under different flight conditions. Several conclusions can be obtained: (1) Fuel is the most important heat sink in modern advanced aircrafts with aerodynamic shape and high stealth, but its cooling capacity is restrictively limited by the mass in the tank, making it difficult to satisfy the increasing cooling demand of thermal loads. (2) RA is more suitable for the low-speed aircraft due to its outstanding cooling ability and low temperature in subsonic flight, however, the utilization of RA will inevitably introduce the additional RAHX and lead to an obvious drag penalty. (3) EFA is similar to RA, but its temperature is higher and exergy penalty is smaller. Compared with RA, the advantages of EFA are that it doesn’t affect the aerodynamic and stealth layout and can be used in the parking state. (4) SHX has the smallest exergy penalty as an ambient heat sink, but due to the limitations of the fuselage area, its cooling ability is far from being enough for modern advanced aircraft. (5) EHS’s exergy penalty is huge, but considering that the combustible liquefied gas can provide thrust or power for aircraft when needed, it has great potential in further hypersonic aircraft. 

The estimation models and analysis results presented in this paper can be used to design and optimize the TMS. Firstly, it can be used to guide the design of various heat sinks—how to determine the parameters of the heat sinks, so that they can provide more cooling ability/capacity while producing less exergy penalty. Secondly, the optimal heat sink ratio can be determined according to the cooling abilities/capacities and exergy penalties of different heat sinks to minimize the total cooling-penalty ratio (total cooling capacity/total exergy penalty). Thirdly, during the flight, the on-time estimating of the cooling abilities/capacities can be used to dynamically manage various heat sinks, further avoiding the waste of heat sinks and prolonging the working time of TMS. At last, according to the different temperatures and characteristics of heat sinks, the heat transfer path of TMS can be optimized to make the most efficient utilization of each heat sink.

## Figures and Tables

**Table 1:** Atmosphere parameters at sea level.

Parameters	Symbol & Value
Temperature	$T_{0}=288\;K$
Pressure	$p_{0}=101,325\;\mathrm{Pa}$
Density	$\rho_{0}=1.225\;{\mathrm{kg}/m}^{3}$

**Table 2:** Parameters for the estimating model of “Case 0”. EHS, expendable heat sink; LNG, liquefied natural gas.

Parameters of Heat Sinks	Symbol	Value
Take-off weight	$m_{a}^{\left(t=0\right)}$	30,000
Engine combustion efficiency	$\eta_{c}$	0.90
Engine mechanical efficiency	$\eta_{m}$	0.35
Engine propulsion efficiency	$\eta_{t}$	0.80
Engine duct fan efficiency	$\eta_{f}$	0.64
Lift-drag ratio of engine	*K*	5.00
Bypass ratio of engine	*B*	0.20
Power partition coefficient of engine	*X*	0.10
Air-fuel ratio of engine	θ	18
Initial Fuel Mass	$m_{fuel}^{\left(t=0\right)}$	15,000
Initial Fuel temperature	$T_{fuel}^{\left(t=0\right)}$	30 °C
Threshold temperature of Fuel	$T_{fuel,threshold}$	80 °C
Special heat of fuel	$c_{p,fuel}$	2010
Combustion value of fuel	$\phi_{fuel}$	$4.3\times 10^{7}$
Tank Surface Area	$A_{tank}$	10
Heat trans coefficient from tank to skin	$k_{tank}$	20
Emissivity of the skin	$\varepsilon_{skin}$	0.7
Initial EHS Mass	$m_{EHS}^{\left(t=0\right)}$	100
Substance of EHS	LNG	

**Table 3:** Parameters of RAHX, FDHX, SHX in “Case 0”. HX, heat exchanger; RAHX, ram air heat exchanger; FDHX, fan duct heat exchanger; SHX, skin heat exchanger.

HX Type	Inlet Area/Skin Area	HX Mass Density	Heat Transfer Area Density	Heat Transfer Coefficient	Heat Transfer Efficiency	Velocity Loss Coefficient
RAHX	$A_{in,RA}=0.02$	$\rho_{RAHX}=600$	$\alpha_{RAHX}=1000$	$k_{RAHX}=200$	$\eta_{RAHX}=80\%$	$\xi_{RA}=0.2$
FDHX	$A_{in,EFA}=0.02$	$\rho_{FDHX}=500$	$\alpha_{FDHX}=800$	$k_{FDHX}=200$	$\eta_{FDHX}=60\%$	$\xi_{EFA}=0.1$
SHX	$A_{skin}=20$	$\rho_{SHX}=300$	$\alpha_{SHX}=600$	$k_{SHX}=120$	$\eta_{SHX}=80\%$	---

**Table 4:** The ram inlet size of Case 1~5.

Case 1	Case 2	Case 3	Case 4	Case 5
$A_{in,RA}=0.01$	$A_{in,RA}=0.02$	$A_{in,RA}=0.04$	$A_{in,RA}=0.07$	$A_{in,RA}=0.10$

**Table 5:** The FDHX frontal size of Cases 6~10.

Case 6	Case 7	Case 8	Case 9	Case 10
$A_{in,EFA}=0.01$	$A_{in,EFA}=0.02$	$A_{in,EFA}=0.04$	$A_{in,EFA}=0.07$	$A_{in,EFA}=0.10$

**Table 6:** The heat transfer efficiency of SHX of Cases 11~15.

Case 11	Case 12	Case 13	Case 14	Case 15
$\eta_{SHX}=50\%$	$\eta_{SHX}=60\%$	$\eta_{SHX}=70\%$	$\eta_{SHX}=80\%$	$\eta_{SHX}=90\%$

**Table 7:** The case design of Cases 16~25.

Case	Case 16	Case 17	Case 18	Case 19	Case 20	Case 21	Case 22	Case 23	Case 24	Case 25
Substance	LNG	LNG	LNG	LNG	LNG	LA	LA	LA	LA	LA
Initial mass	20	50	100	200	300	20	50	100	200	300

**Table 8:** The cooling ability of each heat sink in different flight Mach number.

	Fuel	RA	EFA	SHX	Total
Cruise in Ma 1.8	189.1 kW	0	0	0	189.1 kW
Cruise in Ma 1.2	117.5 kW	57.4 kW	19.8 kW	32.0 kW	226.7 kW
Cruise in Ma 0.9	105.9 kW	157.0 kW	78.1 kW	53.6 kW	394.6 kW

## Nomenclature

**Symbol**		**Subscript**	
*q*	Cooling ability (W)	*a*	Aircraft
*Q*	Cooling capacity (J)	*c*	Combustion
m	Mass (kg)	*m*	Mechanical
$\dot{m}$	Mass flow rate (kg/s)	*t*	Thrust & Propulsive
T	Temperature (K)	*f*	Engine duct fan
*E*	Energy (J)	*k*	Kinetic energy
$\dot{E}$	Energy change rate (W)	*p*	Potential energy
*Ex*	Exergy penalty (J)	max	Maximum
$\dot{E}x$	Exergy penalty rate (W)	min	Minimum
*P*	Power (W)	*d*	Drag
η	efficiency	*w*	Weight
v	Velocity (m/s)	∞	Ambient
Ma	Mach number of aircraft	*0*	Ambient with sea level
H	Altitude of aircraft (m)	ex	Total temperature/pressure
*D*	Drag force	*in*	Air inlet
*A*	Area (m^2^)	*core*	Core of engine
*p*	Pressure (Pa)	*st*	Storage
*t*	Time (s)		
*k*	Heat trans coefficient (W/m^2^K)	**Abbreviations**	
ρ	Density (kg/m^3^)	TMS	Thermal management system
α	Area density (m^2^/m^3^)	HX	Heat exchanger
ϕ	Combustion value (J/kg)	RA	Ram air
$c_{p}$	Specific heat capacity (J/kg K)	RAHX	Ram air heat exchanger
λ	Thermal conductivity (W/m K)	EFA	Engine fan air
μ	Kinetic viscosity (s/m^2^)	FDHX	Fan duct heat exchanger
*h*	Specific enthalpy (J/kg)	SHX	Skin heat exchanger
ξ	Velocity loss coefficient	EHS	Expendable heat sink
*K*	Lift-drag ratio of the aircraft	LA	Liquid ammonia
B	Bypass ratio of engine	LNG	Liquid nature gas
X	Power partition coefficient	ECS	Environment control system
$w_{b}$	Power to per kg bypass stream	HTR	Heat transfer rate
θ	Air-fuel ratio of engine	PAO	Poly Alpha Olefin
ε	Surface emissivity		
χ	Cooling-penalty ratio		
**Constant**			
R	Gas constant	8.314 J/mol K	
g	Acceleration of gravity	9.807 m/s^2^	
σ	Stefan-Boltzmann constant	$5.67\times 10^{-8}{W/m}^{2}K^{4}$	
γ	Ambient temperature lapse rate	0.0065 K/m	
